# miR-197, miR-101, and miR-143 and Pro-Inflammatory Cytokines in Migraine

**DOI:** 10.3390/jcm14186410

**Published:** 2025-09-11

**Authors:** Roberto Carlos Rosales-Gómez, Beatriz Teresita Martín-Márquez, Alvaro Jovanny Tovar-Cuevas, Omar Cárdenas-Saenz, Patricia Orozco-Puga, Milton Omar Guzmán-Ornelas, Nathan Alejandro Peña-Dueñas, Flavio Sandoval-García, Daniela Ortiz-Ríos, Mariana Chávez-Tostado, Diana Mercedes Hernández-Corona, Miriam Méndez-del Villar, Fernanda-Isadora Corona-Meraz

**Affiliations:** 1 Centro de Investigación Multidisciplinario en Salud, Departamento de Ciencias Biomédicas, Centro Universitario de Tonalá, Universidad de Guadalajara, Tonalá 45425, Mexico; roberto.rosales@academicos.udg.mx (R.C.R.-G.);; 2 Instituto de Investigación en Reumatología y Sistema Músculo Esquelético, Departamento de Biología Molecular, Centro Universitario de Ciencias de la Salud, Universidad de Guadalajara, Guadalajara 44340, Mexico; beatriz.martin@academicos.udg.mx (B.T.M.-M.);; 3 UDG-CA-703, “Inmunología y Reumatología”, Departamento de Biología Molecular y Genómica, Centro Universitario de Ciencias de la Salud, Universidad de Guadalajara, Guadalajara 44340, Mexico; 4 Servicio de Neurología del Hospital Civil “Fray Antonio Alcalde”, Departamento de Neurología, Guadalajara 44280, Mexico; 5 UDG-CA-1096, “Ciencias de la Nutrición y Procesos Moleculares del Metabolismo”, Departamento de Ciencias Biomédicas, Centro Universitario de Tonalá, Universidad de Guadalajara, Tonalá 45425, Mexico; 6 Departamento de Reproducción Humana, Crecimiento y Desarrollo Infantil, Centro Universitario de Ciencias de la Salud, Universidad de Guadalajara, Guadalajara 44340, Mexico

**Keywords:** microRNAs, cytokines, inflammation, pain, bioinformatic

## Abstract

**Background**: Migraine is a disabling neurological disorder where the release of neuropeptides and a local and systemic proinflammatory state prevail. MicroRNAs (miRs) are epigenetic regulators that control the expression of genes involved in inflammation, neovascularization, and pain-related processes. Cytokines mediate the inflammatory state, while miRs can modulate their expression. **Methods:** This is an analytical and observational study in which subjects with a diagnosis of chronic and episodic migraine and healthy controls were recruited, and the migraine patients were classified by episodic or chronic migraine, as well as with or without aura. Cytokines were measured using the ELISA technique, and the microRNAs hsa-miR-197-3p, hsa-miR-101-3p, and hsa-miR-143-3p were evaluated using qPCR methodology. We also utilized bioinformatic tools, such as miRBase, TargetScan, miRNet, and miRPath, to analyze the interactions and pathways involved. **Results:** Our findings revealed that hsa-miR-197-3p is elevated in patients without aura (29.91 ± 11.14 with aura vs. 81.10 ± 53.85 without aura, RU; *p* = 0.021), whereas hsa-miR-143-3p is elevated in episodic migraine (0.0639 ± 0.0227 in EM vs. 0.0308 ± 0.0174, RU *p* = 0.011). Furthermore, we found higher levels of IL-17 (9.46 ± 1.06 in CM vs. 7.61 ± 2.12 in EM, *p* = 0.030), IL-6 (4.95 ± 2.84 in CM vs. 1.52 ± 0.98 non-migraine subjects, *p* = 0.016), and TNFα in chronic migraine patients (0.46 ± 0.24 in CM vs. 0.20 ± 0.05 in non-migraine, *p* = 0.011 and vs. 0.20 ± 0.13 in EM, *p* = 0.016). **Conclusions**: Inflammation is present in migraine regardless of the clinical characteristics of the patients, although it may be accentuated in chronic migraine. Our preliminary findings suggest a potential role for peripheral inflammatory markers, including specific microRNAs (miR-197, miR-101, and miR-143) and cytokines (TNF-α, IL-6, and IL-17A), in the pathophysiology of migraine. These results, although limited by sample size and cross-sectional design, highlight molecular pathways that warrant further investigation.

## 1. Introduction

Migraine is a common and disabling neurological disorder characterized by recurrent attacks of moderate to severe pulsating headaches and is associated with symptoms such as nausea, vomiting, and hypersensitivity to light and sound [1]. According to the International Classification of Headache Disorders (ICDH-3), migraine is classified into two main types: migraine with and without aura. In addition, a clinical form known as chronic migraine (CM) is recognized, defined as headache occurring on fifteen or more days per month for more than three months, with at least eight of those days having migraine features [2]. However, in clinical practice, patients are evaluated through an interview that assesses their level of disability. The Headache Impact Test 6 (HIT-6) was designed to gather information on the impact of pain on a patient’s life for improved management and diagnosis [3].

In the context of the pathophysiological understanding of migraine, the vascular perspective, as part of a neurovascular and neuroinflammatory model, involves the activation of the trigeminovascular system, the release of neuropeptides, and a local and systemic proinflammatory state [4]. Nevertheless, the inflammatory state is mediated by cytokines, which can be produced by various cells but are primarily generated by the cells of the immune system and neurovascular cells.

Furthermore, variations in clinical presentation and treatment response among patients suggest the involvement of epigenetic mechanisms, including post-transcriptional regulation of genes by microRNAs (miRs) [5]. MiRs are small, non-coding RNA molecules that participate in the regulation of gene expression, typically through translation inhibition or mRNA degradation. Their expression profile has been associated with multiple neurological diseases, including migraine [6]. Their expression can be influenced by environmental, inflammatory, and hormonal factors, and they have been proposed to play an important role in neurological diseases and pain sensitization processes [6].

Different studies have reported the expression of various microRNAs in patients with chronic and episodic migraine (EM), as well as in those with and without aura, which may suggest their role as specific biomarkers [7,8].

However, the miRs that we evaluated in this study have been assessed under similar conditions; for example, a study investigated the effect of miR-197-3p on the regulation of the innate inflammatory response in autoinflammatory diseases, showing that its overexpression reduces cytokine production by targeting the IL-1R1 receptor (IL1R1) and attenuating the activation of caspase-1 and IL-1β [9]. While in the field of migraine, miR-197-3p has only been identified as one of the miRNAs with altered expression in patients with chronic migraine in an exploratory study [10]. However, its specific role in differentiating between episodic and chronic migraine remains to be described.

On the other hand, miR-101-3p has been shown to play a role in neuroinflammation and synaptic plasticity, particularly in the context of migraine. It can modulate the expression of genes involved in inflammatory signaling, such as cytokine and toll-like receptor pathways, which are known to contribute to migraine attacks [11,12]. In another way, miR-101-3p may influence microglial activation, thereby affecting the inflammatory milieu in the brain and contributing to migraine chronification [13]. Given its involvement in key pathways related to migraine, miR-101-3p holds potential as a biomarker for diagnosing and monitoring the disease. Its expression levels could provide insights into the inflammatory and synaptic changes occurring during migraine episodes [14].

Finally, miR-143-3p has been implicated in the modulation of peripheral inflammatory and nociceptive pathways. Consistent data reports show that decreased miR-143-3p is associated with inflammatory conditions and chronic pain. This evidence suggests that miR-143 dysregulation may occur primarily in chronic migraine, facilitating nociceptive hyperexcitability [15]. Furthermore, miR-143-3p, along with other miRNAs, has been identified as a potential epigenetic biomarker for migraine, given its differential expression in response to migraine treatments. This highlights its utility in predicting treatment response and understanding the underlying pathophysiology of migraine [5,16]. Together, the three miRs converge in regulating inflammation and pain.

Additionally, cytokines circulating in peripheral blood have been shown to be important peripheral biomarkers in various pathologies. Classically, TNFα and IL-6 have been associated in literature with migraine, as they are central mediators of both systemic and neurogenic inflammation. Indeed, TNFα can sensitize nociceptors and induce pain, while IL-6 is elevated in some migraine patients, especially during migraine attacks [17]. However, the findings have not been forceful, and it has been recognized that the immune response in migraine is complex, involving more molecular components. For its part, IL-17A is a cytokine produced mainly by Th17 lymphocytes and has a highly proinflammatory effect, since it participates in autoimmune and degenerative diseases of the central nervous system (CNS) [18].

However, studies on cytokines in migraine have reported variable results. Some studies found elevated inflammatory markers in migraine, while others showed no differences or found only subtle interictal changes [19].

This inconsistency raises the question of the extent to which systemic inflammation contributes to migraine and whether a reproducible signal can be detected in blood.

Thus, the purpose of this study was to determine whether there are significant differences in the expressions of peripheral microRNAs miR-197-3p, miR-101-3p, and miR-143-3p, as well as in the levels of proinflammatory cytokines (TNFα, IL-6, and IL-17A), between migraine patients and healthy controls and to explain how these biomarkers relate to the severity and clinical impact of migraine.

## 2. Materials and Methods

### 2.1. Study Design and Participant Selection

This is an analytical and observational study in which subjects with a diagnosis of chronic and episodic migraine were recruited according to the International Classification of Headache Disorders criteria (ICDH-3) [2], who agreed to participate in the study through informed consent.

The healthy subjects in the control group were recruited from the general population in the western state of Jalisco, Mexico, through community outreach and invitations from hospital staff. They were evaluated by a neurologist via a structured interview and a brief neurological examination to rule out subclinical symptoms.

On the other hand, patients with migraine were classified according to aura manifestation (17 with aura vs. 12 without) and by the frequency of the episodes (20 EM and 9 with CM). Also, migraine patients only took NSAID medications and were in the interictal period; therefore, the sample was taken on a day when they had not taken any NSAIDs. However, the NSAIDs taken by patients during migraine attacks were ibuprofen (400 mg/day), ketorolac (10–30 mg/day), and paracetamol (500 mg/day).

Each patient underwent a comprehensive evaluation by a neurologist specializing in headaches. The diagnosis and classification of migraine were confirmed based on ICDH-3 criteria. Study subjects were interviewed to collect demographic data and migraine history, including disease duration (years since diagnosis), attack frequency (number of migraine days per month), average attack duration, and typical pain intensity and disabling conditions with the HIT-6.

### 2.2. Population Criteria

Inclusion criteria included the absence of a previous diagnosis of migraine or other primary or secondary headache disorders, as well as the absence of a family history of migraine in first-degree relatives. None of the healthy subjects reported regular alcohol consumption, smoking, recent infections, or vaccinations. The women participating in the control group were non-pregnant, premenopausal, and not using hormonal contraceptives at the time of their evaluation.

Additionally, migraine patients and healthy subjects were carefully selected to avoid confounding factors associated with systemic inflammation or other conditions that could alter miRNA or cytokine levels. Thus, study subjects were excluded if they had inflammatory or autoimmune diseases such as rheumatoid arthritis, systemic lupus erythematosus, active infections (in the respiratory or urinary tract, for example), or inflammatory bowel disease. Furthermore, study participants were excluded if they were taking systemic corticosteroids, immunosuppressive drugs, or biological treatments such as TNFα inhibitors or antiglobulin therapies; were pregnant; were pre- or menopausal; or used hormonal contraceptives at the time of evaluation [20]. Additionally, participants were excluded if they had elevated markers of acute inflammation, such as an erythrocyte sedimentation rate (ESR) [21], concomitant neurological disorders that could influence inflammatory, neurodegenerative processes, or other chronic pain syndromes other than migraine (e.g., fibromyalgia or chronic peripheral neuropathic pain) [15].

### 2.3. Plasma and Serum Collection

Whole blood was collected from all subjects using the BD Vacutainer^®^ system (Becton Dickinson and Company, Franklin Lakes, NJ 07417, USA) in EDTA tubes and EDTA-free tubes by antecubital venipuncture, performed in the morning after an overnight fast to minimize circadian and dietary variations in biomarker levels. The samples were centrifuged at 1500 RFC at room temperature to obtain plasma and serum, which were stored at −80 °C and −20 °C (respectively) until prosecution. An aliquot of plasma from each participant was reserved for microRNA analysis, while serum aliquots were used for cytokine analysis.

### 2.4. Headache Impact Test 6

Headache impact assessment was performed using the 6-item Headache Impact Test (HIT-6). HIT-6 is a valid tool to discriminate the severity and impact of life in patients with episodic and chronic migraine. This tool was validated by Yang et al. and demonstrated high reliability in internal agreement among patients with migraine, translated and adapted for cultural understanding among participants in the state of Jalisco, Mexico [22]. This instrument measures the impact of headaches on patients’ quality of life, as well as the severity of associated pain. The Spanish version used was previously validated in the Mexican population, ensuring its cultural appropriateness for participants from the state of Jalisco, Mexico. The HIT-6 consists of six questions, each with five Likert-type response options that assess the frequency of symptoms or perceived limitations (“never”, “rarely”, “sometimes”, “very often”, and “always”). Each response is scored out of 6, 8, 10, 11, or 13 points, respectively (from “never” to “always”), yielding a total score ranging from 36 (minimal impact) to 78 (maximum impact). A higher score reflects a greater impact of the headache on the patient’s life. The questionnaire was administered individually by neurologists at the hospital during each subject’s evaluation visit, ensuring consistency in the administration of the test [23].

### 2.5. Extraction and Reverse Transcription of microRNAs from Blood Plasma

The plasma samples obtained were refrigerated and then allowed to return to room temperature to facilitate gradual thawing. They were then treated with the miRNeasy serum/plasma extraction and purification kit (Qiagen, Venlo, The Netherlands, cat. no. 217184,), which combines a phenol/guanidine-based lysing reagent and silica membranes for purification. The extracted samples were stored at −80 °C until reverse transcription.

Reverse transcription was performed using the cDNA synthesis kit, according to the Taqman™ Advanced protocol (Applied Biosystems, Foster City, CA, USA, cat. no. A28007), which includes four preparation steps: poly-A tailing reaction, adapter ligation reaction, reverse transcription, and miR-Amp reaction.

### 2.6. Expressions of microRNAs hsa-miR-197-3p, hsa-miR-101-3p, and hsa-miR-143-3p

Expression was determined by qPCR using cDNA samples on the QuantStudio ™ equipment (Thermo Fisher, Waltham, MA, 02451 USA). Likewise, TaqMan ™ Fast Advanced Master Mix (Cat. No. A25576) and the TaqMan^®^ Fast Advanced miRNA Assays from Thermo Fisher´s, Applied Biosystems (Cat. no. A25576) were used, with hsa-miR-320a-3p as endogenous control and the following targets: hsa-miR-197-3p, has-miR-101-3p, and hsa-miR-143-3p. The expression of each miRNA was determined using the comparative ∆CT method against the endogenous control. Relative expression units were calculated using the 2^−∆CT^ method, where ∆CT = Ct target − Ct_miR-320a-3p. This transformation allowed us to obtain normalized relative expression values, which were used in comparative analyses between groups [24].

### 2.7. Quantification of Serum Levels of IL-17A, IL-6, and TNFα

Serum samples were thawed and then homogenized. Subsequently, serum concentrations of the proinflammatory cytokines IL-17A, IL-6, and TNFα were measured using commercially available enzyme-linked immunosorbent assay (ELISA) kits (Quantikine^®^ ELISAs, R&D Systems, Minneapolis, MN 55413, USA). Specifically, we used the Human IL-17 Quantikine ELISA kit (Cat. no. D1700), the Human IL-6 Quantikine ELISA kit (Cat. no. D6050), and the Human TNFα Quantikine ELISA kit (Cat. no. DTA00D). All assays were performed strictly by the manufacturer’s instructions. Samples were run in duplicate wells to ensure accuracy.

### 2.8. Network Analysis

The databases https://www.mirbase.org/ (miRbase) and https://www.targetscan.org/ (TargetScan), as well as https://www.mirnet.ca/miRNet, (miRNet), were used to search for genes and miR interactions, using the miRs for the analysis, searching for all the genes that they can regulate and the interaction score that they have. Common genes can also be reviewed through a cross-analysis through a statistical model that predicts the effects of microRNAs binding to canonical sites, which considers the following characteristics: site type, 3′-supplementary pairing, local AU content, distance from the closest 3′-UTR, target-site abundance (TA) within the transcriptome, and stronger predicted seed-pairing stability (SPS) [25].

In addition, each miR was analyzed individually using https://dianalab.e-ce.uth.gr/ (miRPath V.4) to analyze the signaling pathways involved, which used a functional enrichment analysis through Gene Ontology (GO) and KEGG terms in the predictive or experimental context [26]. All revisions and analyses were carried out between 10 March 2025 and 31 April 2025.

### 2.9. Statistical Analysis

All relative expression values (2^−∆CT^) were statistically analyzed in their logarithmic form to meet the assumptions of normality and homogeneity of variance and to avoid bias due to asymmetric distribution. All data were analyzed using IBM SPSS statistics version 23 (IMB SPSS Software, Armonk, NY, USA). The statistical differences were analyzed using the U Mann–Whitney, Kruskal–Wallis, and Spearman tests. Data considered significant at *p* < 0.05 were expressed as the mean or median and standard error of the mean.

## 3. Results

### 3.1. Demographic and Clinical Characteristics in Patients and Controls

This study enrolled 29 migraine patients and 24 clinically healthy subjects who were predominantly women (80% and 72%, respectively), with average ages of 35 ± 11 and 45 ± 9 years (respectively). In addition, patients with chronic and episodic migraine had the following demographic and clinical characteristics, which are shown in Table 1.

### 3.2. miR-143 Is Highly Expressed in Patients with Migraine

**Figure 1:**
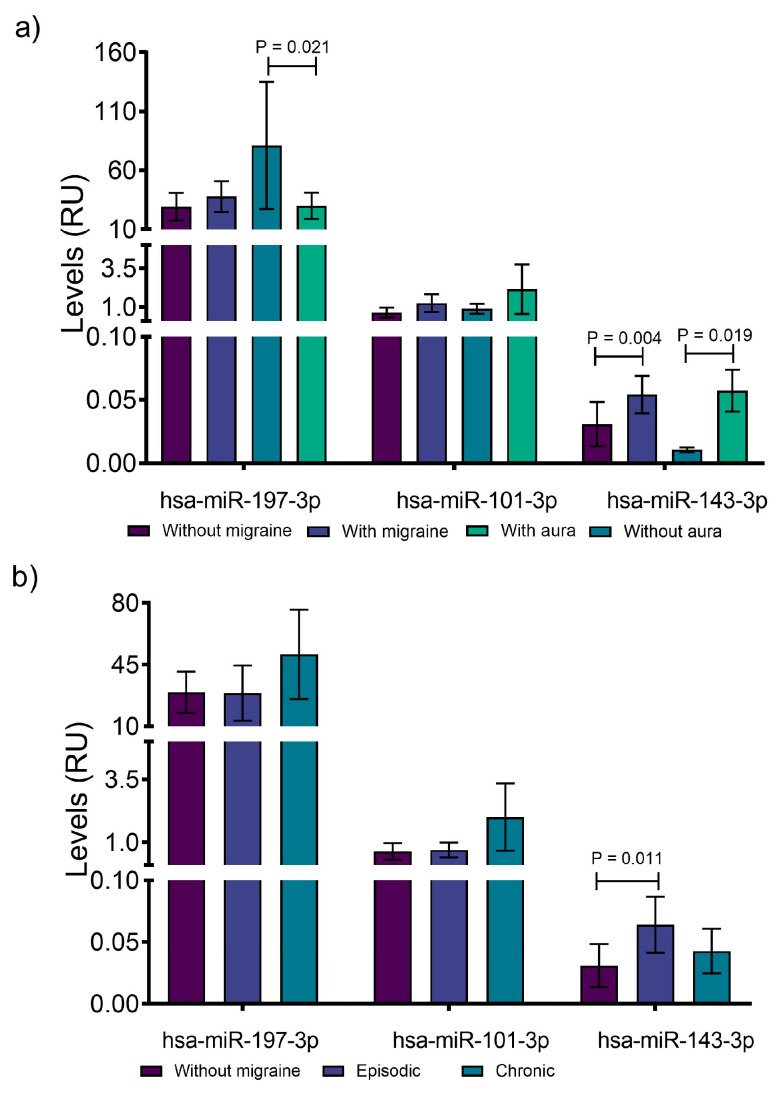
Expression levels of miR-197, miR-101, and miR-143 in the plasma of migraine patients. (**a**) Comparative expressions of the studied microRNAs and their relationship with migraine and the presence or absence of aura. (**b**) Comparative expression of microRNAs with respect to headache frequency. The analysis was carried out through Kruskal–Wallis tests. RU: relative units.

We observed that hsa-miR-197-3p was more highly expressed in patients who reported not having aura symptoms (29.91 ± 11.14 with aura vs. 81.10 ± 53.85 without aura, RU; *p* = 0.021). On the contrary, hsa-miR-143-3p showed higher levels in patients with aura (0.0572 ± 0.0165 with aura vs. 0.0106 ± 0.0019 without aura, RU *p* = 0.019) and in the patients with EM vs. without migraine (0.0639 ± 0.0227, RU *p* = 0.011), in addition to showing an increase in migraineurs in general (0.0542 ± 0.0148 with migraine vs. 0.0308 ± 0.0174 without migraine, RU *p* = 0.004). On the other hand, hsa-miR-101-3p did not show changes when analyzed in this way (Figure 1).

### 3.3. In Chronic Migraine, There Are Higher Levels of Pro-Inflammatory Cytokines and Association with miR-101-3p

**Figure 2:**
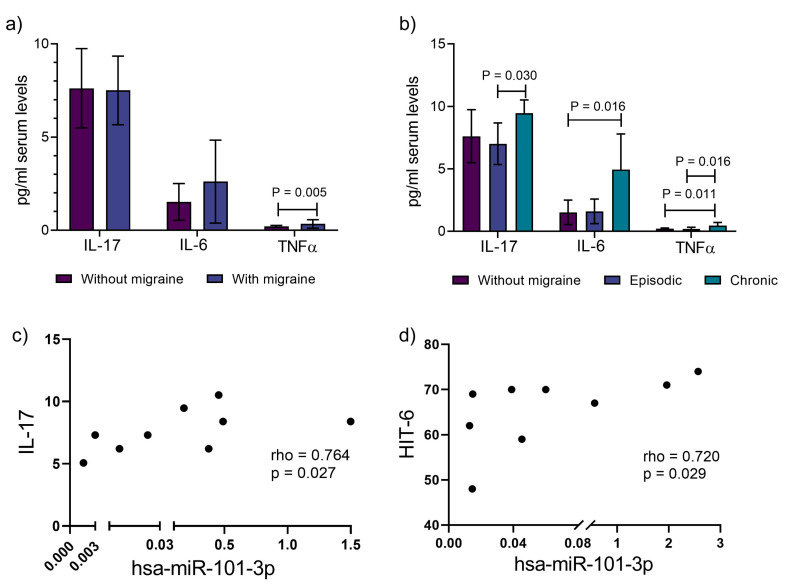
Proinflammatory cytokines and the correlation of hsa-miR-101-3p with the presence and clinical severity of migraine. (**a**) Levels of cytokines in migraine and healthy subjects, (**b**) levels of cytokines in episodic, chronic migraine, and healthy subjects, (**c**) miR-101 correlation with IL-17 in chronic migraine patients, (**d**) miR-101 correlation with HIT-6 score in chronic migraine patients. IL: interleukin. TNFα: tumor necrosis factor alpha. HIT-6: Headache Impact Test 6. Data were analyzed with U Mann–Whitney and Kruskal–Wallis tests, and rho of Spearman was carried out for correlations.

We evaluated three cytokines recognized for their role in inflammation. Interestingly, we observed that TNFα was increased in migraine patients (0.34 ± 0.23 vs. 0.20 ± 0.05; *p* = 0.005), and when the chronicity of events classified these, all three cytokines were increased in CM patients. For example, IL-17 was higher in CM vs. EM (9.46 ± 1.06 vs. 7.61 ± 2.12, *p* = 0.030), IL-6 in CM vs. non-migraine subjects (4.95 ± 2.84 vs. 1.52 ± 0.98, *p* = 0.016), and TNFα was higher in CM vs. non-migraine and EM subjects (0.46 ± 0.24 vs. 0.20 ± 0.05, *p* = 0.011 and vs. 0.20 ± 0.13, *p* = 0.016) (Figure 2a,b). This was not observed in patients classified as having migraine without aura, where no differences were noted.

Considering the relevance of CM to the elevation of inflammatory cytokines, we analyzed whether the miRs studied were involved with these cytokines or the disability score presented in the patients (HIT-6). We found that in CM, miR-101-3p was positively associated with IL-17, a cytokine recognized for its role in inflammatory events (Figure 2c). Additionally, among the miRs studied, this one showed a correlation with the levels of the HIT-6 score (Figure 2d).

### 3.4. miR-197, miR-101, and miR-143 Are Related to Pain and Inflammation Genes

**Figure 3:**
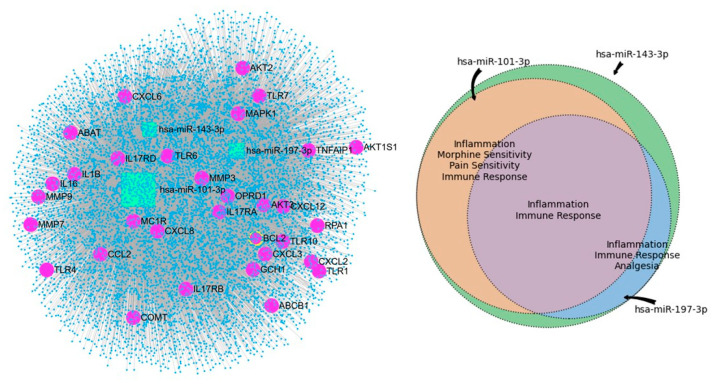
Interaction network between microRNAs and target genes associated with inflammation and pain. The network was constructed using network analysis using integrated data from miRBase, TargetScan, and miRNet (using GO terminology and the KEGG pathway). The microRNAs hsa-miR-143-3p, hsa-miR-101-3p, and hsa-miR-197-3p (light-blue nodes) and their main target genes (magenta nodes), including IL1B (interleukin-1 beta), IL6 (interleukin-6), CXCL8 (IL-8), CXCL12, CCL2 (MCP-1), MMP9 (matrix metalloproteinase-9), TLR4, TLR6, TLR7, AKT2, and MAPK1, are shown. Node sizes are proportional to the degree of connectivity (number of interactions), and edges represent validated experimental interactions. The Venn diagram shows the overlaps of functional gene sets associated with hsa-miR-101-3p, hsa-miR-143-3p, and hsa-miR-197-3p.

To understand the role of inflammation in migraines, a network analysis was performed to review which genes might also be regulated for these miRs. Analysis of the miR–target gene interaction network demonstrated a highly connected architecture for the microRNAs hsa-miR-143-3p, hsa-miR-101-3p, and hsa-miR-197-3p. Figure 3 shows the binding of microRNAs to key genes associated with inflammatory signaling and nociception, including TLR4, TLR6, TLR7, IL1B, IL6, IL-17R, CXCL8, CCL2, and MMP9, all of which are implicated in microglial activation, amplification of the innate immune response, and central sensitization. Additionally, genes that modulate the AKT/MAPK pathway (AKT2, MAPK1) and leukocyte chemotaxis (CXCL12, CXCL6) emerged as relevant nodes in the network. The microRNAs studied indicated distinct patterns of functional interaction. The Venn diagram in Figure 3 shows a functional overlap between key molecular processes: inflammation, immune response, pain sensitivity, morphine sensitivity, and analgesia. The three microRNAs share the regulation of genes related to inflammation and immune response. Specifically, hsa-miR-101-3p is also associated with pain and morphine sensitivity and hsa-miR-197-3p with analgesia, while hsa-miR-143-3p is involved in all of the aforementioned processes.

### 3.5. Biological Pathways Modulated by miR-197, miR-101, and miR-143 in Patients with Migraine

Enrichment analysis of biological pathways associated with differentially expressed miRNAs (miR-197-3p, miR-101-3p, and miR-143-3p) (Table 2) estimated significant regulation of multiple pathways involved in the pathophysiology of chronic migraine. Among the most relevant pathways were nervous system development (GO: nervous system development; 134 genes; *p* = 1.07e − 8) and MAPK family signaling (Reactome: MAPK family signaling cascades; 78 genes; *p* = 6.8e − 6), both of which are essential in central sensitization and neuroinflammation mechanisms. Likewise, the PI3K–Akt pathway (71 genes; *p* = 2.94e − 4) and the TGF-β signaling pathway (22 genes; *p* = 0.0101) are important in damage response processes, neuroprotection, and possible regulation of glial activity. Relevant interactions with vascular processes, such as vascular smooth muscle contraction and vasculogenesis (28 and 19 genes, respectively), were also detected, consistent with the findings of neurovascular dysfunction reported in chronic migraine [27]. Finally, the Alzheimer’s disease-associated signaling pathway (74 genes; *p* = 0.00337) stands out for its potential link to shared mechanisms of neurodegeneration and synaptic dysfunction.

## 4. Discussion

Migraine is known for being painful, uncomfortable, and even highly disabling, depending on how frequently these events occur, with chronic migraine with aura being the most disabling of all. However, little is known about how one leads to the other, with the activation of the trigeminovascular system being the most studied, along with the elevation of calcitonin-associated peptides. New epigenetic markers, such as microRNAs, have been studied in various pathologies, including migraine.

Interesting findings include miRs-1307-5p, miR-6810-5p, let-7e, and miR-140-3p, which were found to be elevated during the ictal phase in episodic migraineurs (EM) [28]; miR-155-5p, which modulates microglia polarization and participates in inflammatory processes, however, was found to be elevated in a mouse model of CM, which is supposed to affect neuroinflammation [29]. Thus, inflammation plays an important role in migraine; although it was initially considered primarily a neurovascular disorder, it is now recognized that the inflammatory pathway is closely associated with pain and the elevation of neurotransmitters, such as Calcitonin Gene-Related Peptide and Pituitary Adenylate Cyclase-Activating Peptide [30].

In inflammation, the elevation of cytokines is recurrent, and the migration of immune system cells, once present in the tissue, generates alterations in pain signaling pathways through a positive feedback loop with neuronal cells [31].

Increases in various cytokines have been observed in several studies, although inconsistent results have also been reported [32]. For example, TNF-α and IL-6 are both elevated and remain unchanged, even though these studies differ primarily in the timing of sampling.

In a study conducted by Sarchielli et al., patients without aura were sampled 30 min after the onset of the attack, with time intervals until the attack ended, and it was found that IL-6 and TNFα increased at 2 h, while IL-1β and IL-4 decreased over time [33].

Regarding chronicity, Mansoureh Togha et al. found elevated levels of TNF-α, IL-6, and C-reactive protein in patients with CM, which could lead us to think that as these inflammatory markers increase, migraine tends to become chronic [34]. However, Fahimeh Martami et al. found the highest levels in patients with EM, so it is not yet possible to clarify how inflammation affects migraine progression [35].

Notably, our results showed higher levels of the cytokines TNFα, IL-6, and IL-17 in CM; even TNF-α was shown to be higher in these than in those with EM; therefore, we cannot assume that chronicity is due to the present inflammation, but rather, we hypothesize that the same inflammation, present and constant, precedes chronicity; however, there are key moments when the attacks are so recurrent that it makes it more complicated for the inflammatory environment to return to a basal state.

A good example is IL-17, which is characterized by a gradual increment in chronic inflammatory diseases [36]. However, IL-17 has been poorly evaluated in migraine; for example, in a rat model of migraine induced with NTG, IL-17A was found in the medulla oblongata and associated with neuroinflammation [37], in addition to being elevated in patients with neurodegenerative diseases, directly associating it with neuroinflammation [38], and in others where different treatments were used, it was associated with nano-curcumin and vitamin D3 in patients with episodic migraine, which showed a reduction in IL-17 levels [39,40].

In our study, we found that IL-17 is associated with miR-101, which leads us to suggest that miR-101 may be more closely related to inflammatory pathways and may influence the severity and progression of migraine by affecting microglial activity [10,13]. Additionally, when we performed the bioinformatics analysis, we found that the three miRs we studied are primarily related to the MAPK pathway, which is a key player in the development of inflammation. Moreover, miR-101 has been implicated in regulating inflammatory pathways through its interaction with the MAPK pathway, which is crucial for IL-17A production [41,42].

While the direct effect of miR-101 on IL-17A production in the context of migraine has not been fully elucidated, the role of miR-101 in modulating the inflammatory response through the MAPK pathway suggests a possible indirect influence on IL-17A production and the progression of inflammatory disease in migraine. miR-101 directly targets MKP-1, a phosphatase that inactivates MAPKs, resulting in prolonged activation of MAPK pathways, including p38 and JNK, which are involved in inflammatory responses [41]. Overexpression of miR-101 has been shown to promote inflammation by increasing the levels of proinflammatory cytokines, such as IL-1β, IL-6, and TNF-α, in microglial cells, which are part of the central nervous system’s immune response [43]. However, when the MAPK pathway is activated, it can lead to the production of several proinflammatory cytokines, potentially including IL-17A, although this specific link is not directly established in the context of migraine [44].

IL-17A can cross the blood–brain barrier under pathological conditions and promote the activation of microglia in the CNS. Studies in murine models of induced chronic migraine have shown that high levels of IL-17A in the blood can infiltrate the trigeminal nucleus and trigger central neuroinflammation, activating nociceptive pathways of migraine. Chen et al. reported that blocking IL-17A in this model significantly attenuated glial activation and hyperalgesia, suggesting that IL-17A is the causal factor in the chronification of migraine [37]. These preclinical results strongly suggest that IL-17A is an active mediator in the pathophysiology of migraine. Human studies, particularly in the pediatric population, found that children with migraine had significantly higher serum levels of IL-17A compared to healthy controls, even outside the crisis, and that these levels were associated with an increased risk of migraine (OR ≈ 1.07 per increment, *p* = 0.01). The authors suggest that IL-17A (together with IL-12) may have diagnostic/predictive value in childhood migraine [20].

On the other hand, IL-17A plays a key role in chronic pathologies such as multiple sclerosis, where it contributes to neuronal degeneration via inflammation. Its presence in migraine would be consistent with the hypothesis that a systemic proinflammatory state exists in subgroups of migraine sufferers. Furthermore, IL-17A can induce the production of other cytokines (IL-6, TNF) in endothelial cells and glia, amplifying the inflammatory cascade. The decision to measure it alongside TNF and IL-6 suggests a more comprehensive view: TNFα and IL-6 may reflect acute-phase inflammation or a general immune response, while IL-17A indicates activation of the Th17/autoinflammatory branch.

On the other hand, the relationship between miR-101 expression levels and HIT-6 has not been specifically studied. However, their use as biomarkers could help predict the severity and frequency of migraine episodes. A composite model that integrates miRNA expression and clinical scores has been suggested as a predictive tool to identify individuals at high risk for migraine, potentially improving the accuracy of migraine diagnosis and treatment [28].

Another interesting result is that we found miR-197-3p and miR-143-3p to be increased in migraine, with the former being higher in patients without aura and the latter in patients with aura, particularly those with EM.

In this regard, it has been observed that transfection of miR-197 into immune cells reduces the levels of mediators such as IL-6 and TNFα [45]. This evidence suggests that miR-197 acts as an inflammatory brake, and its dysfunction could lead to an uncontrolled proinflammatory state, while miR-143 was postulated as a marker of response to erenumab in migraine, where it decreased with greater response to the treatment. However, this study did not differentiate between episodic and chronic patients [16].

Rather, miR-143 has been more closely linked to cardiovascular events and cancer because its role as a regulator of cell differentiation and proliferation genes has been better studied [46,47].

Nevertheless, significantly reduced levels of miR-143-3p have been reported in peripheral mononuclear cells in patients with fibromyalgia [15,48], a notable finding, given that fibromyalgia shares similarities with migraine in terms of central sensitization and imbalances in pain mediators. At the functional level, miR-143 plays an analgesic–inflammatory role by downregulating COX-2 (cyclooxygenase-2), an important enzyme in the production of pro-pain prostaglandins, through the degradation of its mRNA. Additionally, miR-143 has been reported to target multiple genes related to pain and inflammation pathways, including TNF-α, interleukins, and MAPK kinases. The reduction of miR-143, therefore, leads to an increase in the expressions of these nociceptive mediators. In animal models, following the induction of inflammation (Freund’s adjuvant injection), a marked decrease in miR-143 was observed in the dorsal ganglia, associated with increased pain [15].

On the other hand, miR-197 and miR-101 have been more involved in cancerous processes, with miR-101 being the most studied [49,50]. However, it is indeed notable that, in addition to their role in inflammation, these miRs have also played a significant role as regulators of pain genes [51].

Liu et al. described that in patients with peripheral neuropathy, low expression of miR-101 was associated with the disinhibition of its target KPNB1 and the subsequent activation of NF-κB. This process contributes to the pathogenesis of pain [52]. At the experimental level, some animal models demonstrated that the increase in miR-101 in microglia can exacerbate hypersensitivity to pain. In rats with chronic nerve injury, overexpression of miR-101 in the spinal cord enhanced inflammatory activation of microglia and increased allodynia [43]. Hence, miR-101 may play a fine-tuning regulatory role in the neural inflammatory response. Its sustained downregulation promotes the activation of proinflammatory pathways (NF-κB), whereas its acute overexpression may also exacerbate the local inflammatory reaction. This dual role highlights the potential importance of miR-101 in chronic migraine.

The expression levels of miR-143 and miR-101 observed in our study suggest that the pathophysiology of migraine qualitatively differs from that of fibromyalgia and neuropathic pain, at least with respect to the dynamics of these genetic regulators. In fibromyalgia, reduced miR-143 expression has been associated with the loss of its anti-inflammatory function, since miR-143 typically suppresses COX-2 and TNF-α [53,54]. In migraine, transient inflammatory pathways are more likely involved, where leukocytes or peripheral tissues induce miR-143 expression to limit inflammation through a compensatory or feedback mechanism, consistent with the episodic nature of the disorder. By contrast, in neuropathies, low expression of miR-101-3p is linked to unchecked NF-κB and the sustained production of inflammatory cytokines in injured nerves [52].

We acknowledge that our study has some limitations. Our modest sample size likely failed to detect subtle changes or limited the statistical power and generalizability of the findings, so we suggest that they be considered with caution. However, the sample size is justified by the clinical nature of migraine and the strict application of inclusion and exclusion criteria. This situation led to reducing the study population of eligible patients to ensure a more homogeneous and valid sample.

It is important to mention that the patients included in the study were treated pharmacologically with acute therapies such as NSAIDs but not with triptans, gepants, or ditans, since the latter tend to have half-lives of at least 24 h [55]. Although NSAIDs in general have much shorter half-lives than triptans, gepants, and ditans, it is important to consider that NSAID consumption may influence proinflammatory cytokine levels. NSAIDs exert their anti-inflammatory effects primarily by inhibiting cyclooxygenase and consequently decreasing prostaglandins, which may indirectly modulate cytokine production. Several studies have documented that NSAID administration is associated with decreases in the concentrations of proinflammatory cytokines, such as interleukin-6 (IL-6), IL-17, and tumor necrosis factor alpha (TNF-α). For example, in patients with ankylosing spondylitis, treatment with NSAIDs significantly reduced serum levels of IL-6, IL-17, and TNF-α [56]. Similarly, in vitro experimental models have shown that NSAIDs can inhibit the production of IL-6 and TNF-α by activated immune cells or tissues [57]. These findings suggest that regular or recent NSAID use tends to attenuate circulating levels of proinflammatory cytokines, which could potentially confound the interpretation of their baseline levels in studies of migraine patients.

Migraine is not a homogeneous entity. There are phenotypes (with vs. without aura, episodic vs. chronic); therefore, our sample size did not allow for robust subgroup analysis. The trends observed in our subgroups and the controversial results reported in previous studies examining inflammatory cytokines in migraine further underscore our caution regarding our findings. We suggest that future studies with larger sample sizes should explore this heterogeneity in cytokine expression, which would allow for a more definitive assessment of the influence of sex, migraine severity, aura status, and other factors.

In this study, our results suggest preliminary data that migraine patients exhibit distinctive peripheral alterations—both at the level of proinflammatory microRNAs and cytokines—supporting the hypothesis that migraine is a disorder with a moderate systemic inflammatory component. We found significant elevations of miR-197, miR-101, and miR-143, along with increased levels of IL-6, TNF-α, and IL-17A, compared to controls, suggesting the existence of an immunomolecular signature associated with migraine in relation with the miR-101/IL-17A pathway and clinical severity (HIT-6), which should be explored in large, longitudinal cohorts.

In this sense, some of the questions that have remained unanswered in the field are related to the lack of validated peripheral biomarkers that aid in the diagnosis or monitoring of migraine activity as a complex neurological disorder [58]. Identifying circulating molecules associated with migraine could shed light on how we understand and manage this disease, but previous findings have been limited and sometimes inconsistent.

These findings, although they should be interpreted with caution, given the aforementioned limitations, contribute a novel piece to the pathophysiological puzzle of migraine. They help fill the knowledge gap regarding peripheral biomarkers in migraine, indicating that it is possible to detect measurable changes in peripheral blood that distinguish migraineurs from non-migraineurs. This opens exciting possibilities for the future, suggesting that we may soon have new tools for diagnosing and predicting migraine.

## 5. Conclusions

Migraine involves detectable inflammatory components in peripheral blood potentially associated with pathogenesis. Elevated levels of miR-197, miR-101, and miR-143, along with proinflammatory cytokines (TNF-α, IL-6, and IL-17A), align with the hypothesis of migraine as an inflammatory process. These findings point to molecular pathways of interest—such as the Th17/miR-101/MAPKs/NF-κB axis—that require further exploration in the pathophysiology of headaches. Our findings present intriguing research perspectives, such as understanding the involvement of IL-17A and related pathways or examining whether the modulation of microRNAs, such as miR-101 or miR-143, influences the frequency and intensity of migraine. However, these conclusions should be interpreted with caution. The study’s cross-sectional design, the relatively small sample size—particularly the limited number of patients with chronic migraine—and the exclusive sampling during the interictal phase restrict the generalizability of our findings. Nonetheless, our data open promising avenues for future research, including the role of IL-17A and associated immune pathways, as well as the potential impact of modulating specific microRNAs, such as miR-101 or miR-143, on migraine frequency and severity.

## Figures and Tables

**Table 1:** Demographic and clinical manifestations in the study population.

	Episodic Migraine (n = 20)	Chronic Migraine (n = 9)	Control (n = 24)
Age (y)	35 ± 11	36 ± 11	45 ± 9
Gender (women), n (%)	20 (100%)	3 (33%)	17 (71%)
BMI (kg/m^2^)	25.7 ± 5.1	27.2 ± 5.2	23.1 ± 1.4
Aura, n (%)	11 (55%)	6 (66%)	NA
Disease duration (y, min-max)	2–35	1–7	NA
Frequency of migraine events (per month)	1–12	15–26	NA
HIT-6 score	65 ± 7	68 ± 2	NA
NSAIDs	Ketorolac, paracetamol, ibuprofen		-

y: years; BMI: body mass index; HIT-6 score: Headache Impact Test-6 score; NSAIDs: nonsteroidal anti-inflammatory drugs.

**Table 2:** Pathways intervened by miRs-197-3p, -101-3p, and 143-3p in humans.

Interaction Pathways of miRs	Target Genes	*p*-Value
MAPK signaling pathway	66	0.000108
PI3K–Akt signaling pathway	71	0.000294
Alzheimer’s disease	74	0.00337
Vascular smooth muscle contraction	28	0.00845
TGF-beta signaling pathway	22	0.0101
Nervous system development	134	1.07 × 10^−8^
MAPK family signaling cascades	78	0.0000068
Nervous system development	103	5.05 × 10^−7^
Vasculogenesis	19	0.0000567

Kegg, Reactome, and Go terms were used in miRPathv4.0.

## Data Availability

Additional information from the data analysis is available on a drive account that the first author has published: https://drive.google.com/drive/folders/1MOECTiXts_uyvUJ_4DRBhjKsZeQ-2I_l?usp=sharing (27 June 2025).

## Abbreviations

The following abbreviations are used in this manuscript:

ICDH-3	International Classification of Headache Disorders
CM	chronic migraine
EM	episodic migraine
HIT-6	Headache Impact Test 6
miRs	microRNAs
CNS	central nervous system
ESR	erythrocyte sedimentation rate
IL1R1	interleukin 1 receptor 1
